# Externalizing traits: Shared causalities for COVID-19 and Alzheimer's dementia using Mendelian randomization analysis

**DOI:** 10.1093/pnasnexus/pgad198

**Published:** 2023-06-15

**Authors:** Haotian Wang, Mingyang Cao, Yingjun Xi, Weijie Cao, Xiaoyu Zhang, Xiaoni Meng, Deqiang Zheng, Lijuan Wu, Wei Wang, Di Liu, Youxin Wang

**Affiliations:** Beijing Key Laboratory of Clinical Epidemiology, School of Public Health, Capital Medical University, Beijing 100069, China; Beijing Key Laboratory of Clinical Epidemiology, School of Public Health, Capital Medical University, Beijing 100069, China; The National Clinical Research Center for Mental Disorders, Beijing Key Laboratory of Mental Disorders & Advanced Innovation Center for Human Brain Protection, Beijing Anding Hospital, Capital Medical University, Beijing 100088, China; Beijing Key Laboratory of Clinical Epidemiology, School of Public Health, Capital Medical University, Beijing 100069, China; Beijing Key Laboratory of Clinical Epidemiology, School of Public Health, Capital Medical University, Beijing 100069, China; Beijing Key Laboratory of Clinical Epidemiology, School of Public Health, Capital Medical University, Beijing 100069, China; Beijing Key Laboratory of Clinical Epidemiology, School of Public Health, Capital Medical University, Beijing 100069, China; Beijing Key Laboratory of Clinical Epidemiology, School of Public Health, Capital Medical University, Beijing 100069, China; Beijing Key Laboratory of Clinical Epidemiology, School of Public Health, Capital Medical University, Beijing 100069, China; Center for Precision Medicine, School of Medical and Health Sciences, Edith Cowan University, Perth 60127, Australia; Center for Biomedical Information Technology, Shenzhen Institutes of Advanced Technology, Chinese Academy of Sciences, Shenzhen, Guangdong 518055, China; Beijing Key Laboratory of Clinical Epidemiology, School of Public Health, Capital Medical University, Beijing 100069, China; Center for Precision Medicine, School of Medical and Health Sciences, Edith Cowan University, Perth 60127, Australia

**Keywords:** externalizing traits, coronavirus disease 2019, Alzheimer's dementia, Mendelian randomization

## Abstract

Externalizing traits have been related with the outcomes of coronavirus disease 2019 (COVID-19) and Alzheimer's dementia (AD); however, whether these associations are causal remains unknown. We used the two-sample Mendelian randomization (MR) approach with more than 200 single-nucleotide polymorphisms (SNPs) for externalizing traits to explore the causal associations of externalizing traits with the risk of COVID-19 (infected COVID-19, hospitalized COVID-19, and severe COVID-19) or AD based on the summary data. The inverse variance–weighted method (IVW) was used to estimate the main effect, followed by several sensitivity analyses. IVW analysis showed significant associations of externalizing traits with COVID-19 infection (odds ratio [OR] = 1.456, 95% confidence interval [95% CI] = 1.224–1.731), hospitalized COVID-19 (OR = 1.970, 95% CI = 1.374–2.826), and AD (OR = 1.077, 95% CI = 1.037–1.119). The results were consistent using weighted median (WM), penalized weighted median (PWM), MR-robust adjusted profile score (MR-RAPS), and leave-one-out sensitivity analyses. Our findings assist in exploring the causal effect of externalizing traits on the pathophysiology of infection and severe infection of COVID-19 and AD. Furthermore, our study provides evidence that shared externalizing traits underpin the two diseases.

**Significance Statement**

This study provides genetic evidence to demonstrate that the externalizing traits were causally related to the increased susceptibility to COVID-19 and AD. The findings highlight the importance to prevent and manage the risk of externalizing traits (e.g. strict regulation measures in cannabis abuse). In addition, our study provides evidence that genetic overlap is identified between externalizing traits and COVID-19 as well as AD and shared externalizing traits underpin the two diseases. Future studies are warranted to explore underlying mechanisms that are responsible for shared causalities.

## Introduction

The externalizing traits referred to behaviors and disorders related to self-regulation, such as opioid use disorder, alcohol use disorder, antisocial behavior, and attention-deficit/hyperactivity disorder. The externalizing liability is highly heritable, with estimates greater than 80% (1, 2), and a recently published genome-wide association study (GWAS) involved in nearly 1.5 million participants has revealed the complex genetic architecture of externalizing traits and identified 579 loci enriched for genes that are expressed in the brain and are involved in the development of the nervous system (3). Their findings suggest that externalizing traits can be defined as a neurodevelopmental trait (4, 5). In addition, the polygenic risk score of externalizing traits was associated with 255 disease phenotypes, such as cardiovascular and cerebrovascular diseases and neurological disorders (3). Such findings point out the important role that externalizing traits play in the emergence of adverse outcomes, indicating that externalizing traits may be a potentially novel and important risk factor.

In recent 3 years, the COVID-19 pandemic has resulted in substantial mortality, morbidity, and economic hardship (6). COVID-19 is a heterogeneous infectious disease whose pathogen results from complex factors such as environmental factors, social genetic factors, and their interactions (7–9). The long COVID-19 can cause sensory loss and even trigger neurological disorders, which is of great global public health concern (10, 11). Meanwhile, Alzheimer's dementia (AD), as the major condition of impaired brain health and the major cause of dementia and disability-adjusted life-years, is a large and growing public health problem due to population aging (12). Interestingly, an increasing number of studies have found that AD and COVID-19 share the same gene loci, that is, OAS1 and APOE ε4, and are genetically regulated with an increased risk for severity (13, 14). Epidemiological observation has demonstrated that COVID-19 is associated with long-term cognitive decline, which is an earlier clinical manifestation of dementia (15). And genetically predicted hospitalized and severe COVID-19 carried an increased risk of AD (16). Furthermore, the evidence supported that COVID-19 and AD shared many predictors, such as age, sex, obesity, type 2 diabetes, and hypertension (17, 18). However, the effect of externalizing traits on COVID-19 and AD remains unknown, and there is an urgent need to further explore the copathogenesis underlying the conditions.

Mendelian randomization (MR) is an alternative and gradually popular instrumental variable (IV) analysis by which genetic variants robustly associated with exposures were used as instrumental variables to infer causality (19–21). The MR approach can overcome bias due to confounders as alleles are randomly assorted into the gametes at conception and reduce bias from reverse causation. In addition, genetic variants were used in the MR approach, which typically affect externalizing traits on a long-term basis. The MR approach based on GWAS–summarized data is an excellent strategy to evaluate causality. Therefore, we performed a standard two-sample MR analysis to infer the causality of externalizing traits with infection and severe infection of COVID-19 and AD based on summary statistics GWAS results of externalizing traits, COVID-19, and AD. Externalizing traits include attention-deficit/hyperactivity disorder, problematic alcohol use, lifetime cannabis use, reverse-coded age at first sexual intercourse, number of sexual partners, general risk tolerance, and lifetime smoking initiation. They shared similar genetic background and common risk factors (3); thus, evaluating the causality of one trait on one outcome might induce unavoidable pleiotropy. This can be done with multivariable MR in which a set of genetic variants is used to predict a set of exposure variables (22, 23). Our study is equivalent to a “multivariable MR” approach, which uses multiple genetic instruments associated with seven traits to simultaneously estimate the causal effect of each of the risk factors on the outcome. Understanding the causality of externalizing traits with the susceptibility and severity of COVID-19 together with prevalent AD implies important public health benefits on disease prevention or complication management.

## Materials and methods

### Study design

In our study, a standard two-sample MR analysis was performed to assess the association of exposure (e.g. externalizing traits) with outcomes (e.g. COVID-19 and AD), in which the IV-exposure and IV-outcome associations were assessed from two samples. Ethics approval or consent to participate was not needed due to the use of summary data from published literature or public databases. Our study followed the “Strengthening the reporting of observational studies in epidemiology using Mendelian randomization (STROBE-MR)” (24, 25), and the checklist of items is shown in Table S1.

**Fig. 1.**
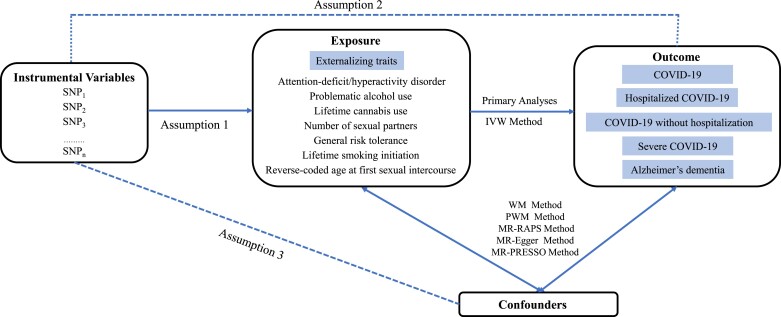
An overview of the MR study design. COVID-19: coronavirus disease 2019; IVW: inverse variance–weighted; MR-RAPS: MR-robust adjusted profile score; MR: Mendelian randomization; PRESSO: pleiotropy residual sum and outlier; PWM: penalized weighted median; WM: weighted median.

As shown in Fig. 1, MR relies on the three main assumptions (26). First, the genetic variants are robustly associated with externalizing traits; second, the genetic variants are independent of multiple factors (vertical pleiotropy) or biological pathways (horizontal pleiotropy) of the externalizing trait–outcome association; third, the genetic variants are independent of the outcomes except through externalizing traits. Of the above assumptions, the most problematic is the second assumption because it is difficult to avoid pleiotropic bias.

### Data sources

We performed a two-sample MR analysis based on summary statistics from the largest available GWAS on externalizing traits, which pooled data from approximately 1.5 million individuals (3). The externalizing traits included seven phenotypes: (i) attention-deficit/hyperactivity disorder, (ii) problematic alcohol use, (iii) lifetime cannabis use, (iv) reverse-coded age at first sexual intercourse, (v) number of sexual partners, (vi) general risk tolerance, and (vii) lifetime smoking initiation. The summarized GWAS data for COVID-19 was downloaded at https://www.covid19hg.org/results/ (27). We used the round five of GWAS meta-analyses for COVID-19 in the European population. Various outcomes of COVID-19 included COVID-19, hospitalized COVID-19, and severe respiratory confirmed COVID-19. The details on the definition of the outcomes were shown in Table S2. The controls were drawn from the general population without the specific phenotype or participants who had COVID-19 without hospitalization, making a total of four comparisons: COVID-19 (*n* = 42,557) vs. general population (*n* = 1,424,707, hereafter COVID-19), hospitalized COVID-19 (*n* = 9,986) vs. general population (*n* = 1,877,672, hereafter hospitalized COVID-19), hospitalized COVID-19 (*n* = 4,829) vs. COVID-19 without hospitalization (*n* = 11,816, hereafter COVID-19 without hospitalization), and severe respiratory confirmed COVID-19 (*n* = 5,105) vs. general population (*n* = 1,383,241, hereafter severe COVID-19). The summarized data for AD was based on the meta-analysis of four GWASs with large sample size, the Psychiatric Genomics Consortium, the International Genomics of Alzheimer's Project (IGAP), the Alzheimer's Disease Sequencing Project (ADSP), and UK Biobank (UKB) (28). Detailed information of the summarized data is shown in Table 1. All the summarized data were European ancestry.

**Table 1.** Basic information of summary statistics data sources in the MR analyses.

Phenotype	Consortium	Total participants or case/controls (*N*)	SNP (*N*)
Externalizing traits	Externalizing Consortium	1,492,085	6,210,733–9,117,721
COVID-19	COVID-19 HGI	42,557/1,424,707	8,738,878
Hospitalized COVID-19	COVID-19 HGI	9,986/1,877,672	8,152,415
COVID-19 without hospitalization^a^	COVID-19 HGI	4,829/11,816	8,375,578
Severe COVID-19	COVID-19 HGI	5,101/1,383,241	9,856,861
AD	IGAP, ADSP, UKB	71,880/383,378	13,367,301

The above summary GWAS data were based on the European population.

a The control is COVID-19 without hospitalization, including laboratory confirmed or self-reported COVID-19; others are the population.

AD: Alzheimer's dementia; ADSP: Alzheimer's Disease Sequencing Project; COVID-19: coronavirus disease 2019; IGAP: International Genomics of Alzheimer's Project; UKB: UK Biobank; MR: Mendelian randomization.

### Selection of genetic variants

The GWAS showed that 579 conditionally and jointly associated (COJO) genetic variants were associated with externalizing traits (*P* < 5 × 10^−8^) (3). In our study, we further filtered out uncorrelated SNPs using linkage disequilibrium (LD) clumping with the lowest *P* value having LD *r*^2^ < 0.001 as final IVs. The LD proxies were defined using 1,000 genomes of European samples. In addition, only SNPs with available SNP–externalizing traits and SNP-COVID-19/AD association data were retained. The detailed information on the selected genetic variants is presented in Table S3, in which 201 SNPs were included for MR analyses of COVID-19 and severe COVID-19, 197 SNPs were included for COVID-19 without hospitalization and hospitalized COVID-19, and 251 SNPs were included for AD. The *R*^2^ [*R*^2^ = 2 × EAF × (1 − EAF)×Beta^2^] in each SNP was estimated, which was summed to calculate the overall *R*^2^ and *F*-statistics [*R*^2^ × (*N* − 2)/(1 − *R*^2^)]. A higher *R*^2^ and *F*-statistic indicate a lower risk of weak IV bias. Detailed information on the IVs is presented in Table S4.

## MR analyses

The inverse variance–weighted (IVW) method was performed as the primary analysis. The Cochran *Q* statistic was calculated to test the heterogeneity between SNPs. The heterogeneity *P* value was less than 0.05, indicating the existence of heterogeneity. The random-effects IVW method was performed if heterogeneity existed; otherwise, the fixed-effects IVW method was used (29). Although IVW can provide precise estimates when all MR assumptions are satisfied, its estimate can be biased under the existence of invalid IVs or pleiotropy. Therefore, we also performed other MR methods to test the robustness from the result of IVW, including weighted median (WM) (30), penalized weighted median (PWM) (30), MR–Egger (31), MR-pleiotropy residual sum and outlier (PRESSO) (32), and MR-robust adjusted profile score (MR-RAPS) (33). We also conducted out leave-one-out sensitivity analyses, in which SNPs were excluded in turn, to explore SNPs that might bias the causal association. In addition, MR–Egger and MR-PRESSO were used to examine the impact of potential pleiotropy on the causal estimates. Odds ratios (ORs) together with their 95% confidence intervals (CIs) were presented for the causal association.

Two-sided *P* < 0.05 was considered nominally suggestive evidence for causal inference, and two-sided *P* < 0.01 was considered statistically significant evidence for a causal association (Bonferroni correction for five outcomes). All data analyses were performed by R version 4.0.3 (https://www.r-project.org/).

## Results

### Causal association of externalizing traits with COVID-19

As shown in Table S5 and Fig. 3, genetically predicted externalizing traits were causally associated with COVID-19 (OR = 1.456, 95% CI = 1.224–1.731, *P* = 2.116 × 10^−5^; *Q* = 250.869, *P*_heterogeneity_ = 0.008) as well as hospitalized COVID-19 (OR= 1.970, 95% CI = 1.374–2.826, *P* = 2.286 × 10^−4^; *Q* = 259.742, *P*_heterogeneity_ = 0.002) by the IVW method. However, no evidence was found in the IVW results to support the causal relationship of externalizing traits with COVID-19 without hospitalization (OR = 1.272, 95% CI = 0.761–2.127, *P* = 0.358; *Q* = 203.102, *P*_heterogeneity_ = 0.349) or severe COVID-19 (OR = 1.556, 95% CI = 0.922–2.628, *P* = 0.098; *Q* = 242.125, *P*_heterogeneity_ = 0.022). According to the heterogeneity test results, a random-effects IVW model was used for COVID-19, hospitalized COVID-19, and severe COVID-19, while fix-effects models were used for COVID-19 without hospitalization (Fig. S1).

**Fig. 2.**
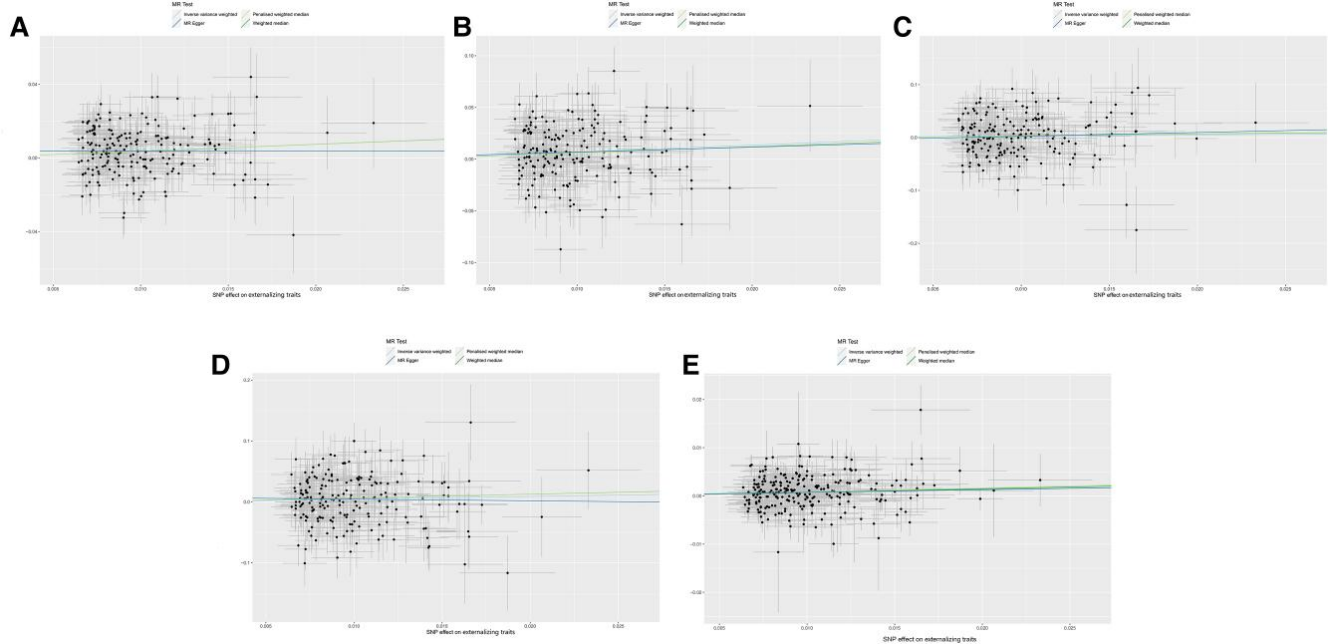
MR scatter plots from externalizing traits to COVID-19 and AD. A) Plot for COVID-19; B) hospitalized COVID-19; C) COVID-19 without hospitalization; D) severe COVID-19; E) AD. Analyses were conducted using the IVW, PWM, WM, and MR–Egger methods. The slope of each line corresponds to the MR effect estimates per method. *X*-axes represent the genetic instrument-externalizing trait associations, and *Y*-axes represent genetic instrument–disease associations from different databases. Each dot denotes the genetic instrument included in the primary MR analyses. AD: Alzheimer's dementia; COVID-19: coronavirus disease 2019; IVW: inverse variance–weighted; MR: Mendelian randomization; PWM: penalized weighted median; WM: weighted medium.

Different MR methods were then employed to appraise the robustness of the above results (Table S6 and Fig. 2). Similar to the findings in the IVW method, genetically predicted externalizing traits presented significant associations with the risk of COVID-19 and hospitalized COVID-19 by applying WM and PWM methods (all *P*s < 0.01). However, the association of externalizing traits with severe COVID-19 or COVID-19 without hospitalization remained negative in the WM, PWM, MR–Egger, MR-PRESSO, and MR-RAPS methods (all *P*s > 0.05). Considering the low *R*^2^ values of SNPs used in this study, we performed MR-RAPS analyses to avoid possible bias caused by low power. There was no evidence to support the causal relationship between externalizing traits and COVID-19 without hospitalization or severe COVID-19 (Tables S5 and S6). In leave-one-out analyses, no outlying genetic variant that had significant influence on estimates was observed for each outcome (Figs. S2–S5).

There's no proof of directional pleiotropy for associations of externalizing traits with COVID-19-related outcomes in MR–Egger regression (all *P*s for intercept > 0.05). In addition, MR-PRESSO analyses found potential pleiotropy in hospitalized COVID-19 and detected two outliers. After correction for outliers, the association with hospitalized COVID-19 was still significant, and the distortion test of MR-PRESSO did not differ significantly in causal estimates before and after outlier corrections (Table S7).

### Causal association of externalizing traits with AD

**Fig. 3.**
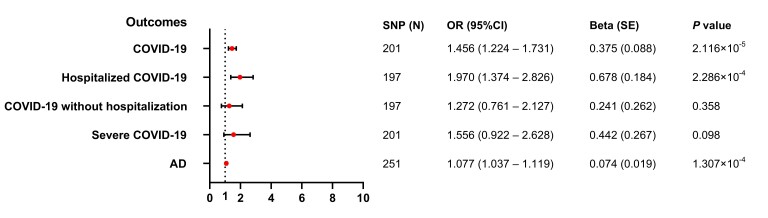
MR forest plots from externalizing traits to COVID-19 and AD. AD: Alzheimer's dementia; COVID-19: coronavirus disease 2019; IVW: inverse variance–weighted; MR: Mendelian randomization; OR: odds ratios; PWM: penalized weighted median; SNP: single-nucleotide poly; WM: weighted medium.

We observed causal evidence of externalizing traits with the risk of AD in a random-effects model of the IVW method (OR = 1.077, 95% CI = 1.037–1.119, *P* = 1.307 × 10^−4^; *Q* = 367.242, *P*_heterogeneity_ = 1.896 × 10^−6^; Table S5 and Fig. 3 and S1). We further conducted sensitivity analyses to verify the reliability of the IVW results. As described in Fig. 2 and Table S6, there were similar estimates with IVW results for externalizing traits with AD using the WM method (OR = 1.076, 95% CI = 1.024–1.131; *P* = 0.004), PWM method (OR = 1.085, 95% CI = 1.032–1.141; *P* = 0.001), and MR-RAPS method (OR = 1.081, 95% CI = 1.046–1.118; *P* = 3.529 × 10^−6^). In addition, we identified outlier SNPs in the MR-PRESSO analysis, and the correction of the outliers did not essentially change the causal results for externalizing traits and AD, which suggested the stability of our results. The leave-one-out analysis indicated that any individual SNP cannot alter the observed causal effect of externalizing traits on AD (Fig. S6).

It is the same as in the analyses of externalizing traits and COVID-19, although there is no evidence of horizontal pleiotropy based on MR–Egger regression as its intercept *P* > 0.05. The *P* value of the global test was less than 0.05, and we found outliers. However, it is unlikely that our results were influenced by horizontal pleiotropy, as no significant difference in causal estimates was observed before and after MR-PRESSO outlier correction (*P* = 0.908; Table S7).

## Discussion

This is the first study using the MR method to explore the effect of externalizing traits on the risk of COVID-19 and AD. Using genetic instruments from the largest available GWAS summary statistics, our study demonstrated that genetically predicted externalizing traits were potentially, yet to be confirmed, causally associated with COVID-19 and AD risk.

Compared with studies on the association of reverse-coded age at first sexual intercourse, the number of sexual partners, and general risk tolerance with the risk of COVID-19 and AD, the associations of attention-deficit/hyperactivity disorder, problematic alcohol use, lifetime cannabis use, and lifetime smoking initiation with the risk of these diseases have been more investigated. Moreover, alcohol use, cannabis use, and smoking initiation were worse during the COVID-19 pandemic (34–37). The causal associations were consistent with previous observations. A previous case report noted that one patient with ADHD developed dementia-like symptoms during the preelderly and elderly stages of life (38). A recent study analyzed a nationwide database of electronic health records of 61 million American adults to explore the correlation between mental disorders and COVID-19 infection. Of note, participants with a recent diagnosis of ADHD had high odds of COVID-19 infection (39). In addition, numerous observational studies have been conducted to investigate the associations of alcohol use, cannabis use, and smoking with COVID-19 or even breakthrough infection (40–42) and have suggested the risk roles played in COVID-19 infection or hospitalization. However, unmeasurable confounding bias and reverse causality inherent in traditional observational studies are also likely to affect the associations.

The results from our MR study are less likely to be biased by confounding or reverse causality than traditional observational studies (26). The causal associations of externalizing traits with COVID-19 and AD have received much attention in recent years (43–47). However, they mainly explored the effect of a single exposure on the outcomes, possibly leading to the bias of weak IVs and, in turn, leading to null findings (44, 45). Of note, our study is equivalent to a “multivariable MR” approach, since we used externalizing traits including seven phenotypes as exposure. Our study greatly improves the power of IVs and then reduces the bias of weak IVs. We found that externalizing traits were causally associated with an increased risk of COVID-19 susceptibility, severity, and AD. Externalizing traits, as a neurodevelopmental trait (4, 5), were prominently associated with increased expression of *CACNA1D* and *PACSIN3*, which were primarily expressed in the brain prenatally. Further investigations are needed to confirm our findings. Understanding these causal relationships will elucidate the underlying mechanisms related to the development of these diseases, and further studies should explore the guiding significance of the causal associations.

Observational studies found that AD can be a risk factor to raise the risk of developing COVID-19 (48), and SARS-CoV-2 might result in an increased susceptibility to neurodegenerative disorders (49, 50). In addition, increasing evidence suggests genetic and pathological relationships between COVID-19 and AD (13, 14, 18), in direct linkage to *APOE4* (51); *APOE4* has been found to be related to COVID-19 infection and mortality (14, 52). Many comorbidities and risk factors are shared in the two diseases (17), indicating that shared pathological processes may be involved in both conditions. Our study provides evidence that shared externalizing traits underpin the two diseases. Future research is required to explore common preventive measures that can modulate ameliorable externalizing traits to prevent or delay the development of COVID-19 and AD.

Our study has notable strengths. Multiple genetic variants from recently published GWASs were used to reduce the bias of weak IVs (3). In addition, the largest GWAS available for each phenotype under investigation provided ample power to detect causal associations. We further conducted a series of sensitivity analyses to test the robustness of our findings. However, several potential limitations are shown in this study. First, the main challenge faced by MR research is pleiotropy, which is mainly because the current MR methods cannot comprehensively evaluate pleiotropy. Although various MR methods were performed in this study to ensure reliability, it can be seen from the difference in pleiotropy test results of MR–Egger and MR-PRESSO that the evaluation of pleiotropy still needs to be further improved, which means that our results also need new MR methods for further verification. Second, the causal of MR–Egger regression was broadly consistent with the conventional MR analysis, in spite of a loss of precision and power, while estimates of weighted median analysis that retained more power than MR–Egger proved remarkably similar to IVW estimates. Although COVID-19 is strongly influenced by exposure to the pathogen and has resulted in a worldwide pandemic, the generalizability of the results is uncertain and may be limited to the European population. Our analyses were based on individual data of mainly the European population, while externalizing traits may vary between different cultural and ethnic populations. Finally, MR analysis assumes a fixed effect of exposure on outcomes, and it is likely to overestimate the effect of exposure intervention on the outcome; therefore, it cannot be assumed to suggest that an intervention to modify the exposure will bring clinical benefits. We hypothesize that interventions to modify externalizing traits need to consider genetic factors.

## Conclusion

This study provides genetic evidence to demonstrate that the externalizing traits were causally related to the increased susceptibility to COVID-19 and AD. In addition, our study provides evidence that shared externalizing traits underpin the two diseases. Future research needs to use genetic evidence to explore effective interventions for COVID-19 and AD with externalizing traits.

## Supplementary Material

pgad198_Supplementary_Data

## Data Availability

The GWAS summary datasets used in this study were from the Externalizing Consortium (PMID: 34446935) for externalizing traits; the COVID-19 host genetics initiative for COVID-19 (); and IGAP, ADSP, and UKB for AD (https://ctg.cncr.nl/software/summary_statistics/).
