# Supplementary material for: Externalizing traits: Shared causalities for COVID-19 and Alzheimer's dementia using Mendelian randomization analysis
Source: PNAS Nexus. 2023 Jun 15;2(6):pgad198. doi: 10.1093/pnasnexus/pgad198 (PMC10287533; doi:10.1093/pnasnexus/pgad198)
Supplement: pgad198_Supplementary_Data [file pgad198_supplementary_data.zip › PNASNEXUS-PNASNEXUS-2022-00978RR-s01.pdf]

**Figure S1. MR funnel plots.**

A: Plot for COVID-19; B: Hospitalized COVID-19; C: COVID-19 without hospitalization; D: Severe COVID-19; E: AD.

Analyses were performed using the IVW and MR–Egger methods. Black dots denote the genetic instruments included in the primary MR analyses.

AD: Alzheimer's dementia; COVID-19: coronavirus disease 2019; IVW: inverse-variance-weighted; MR: Mendelian randomization.

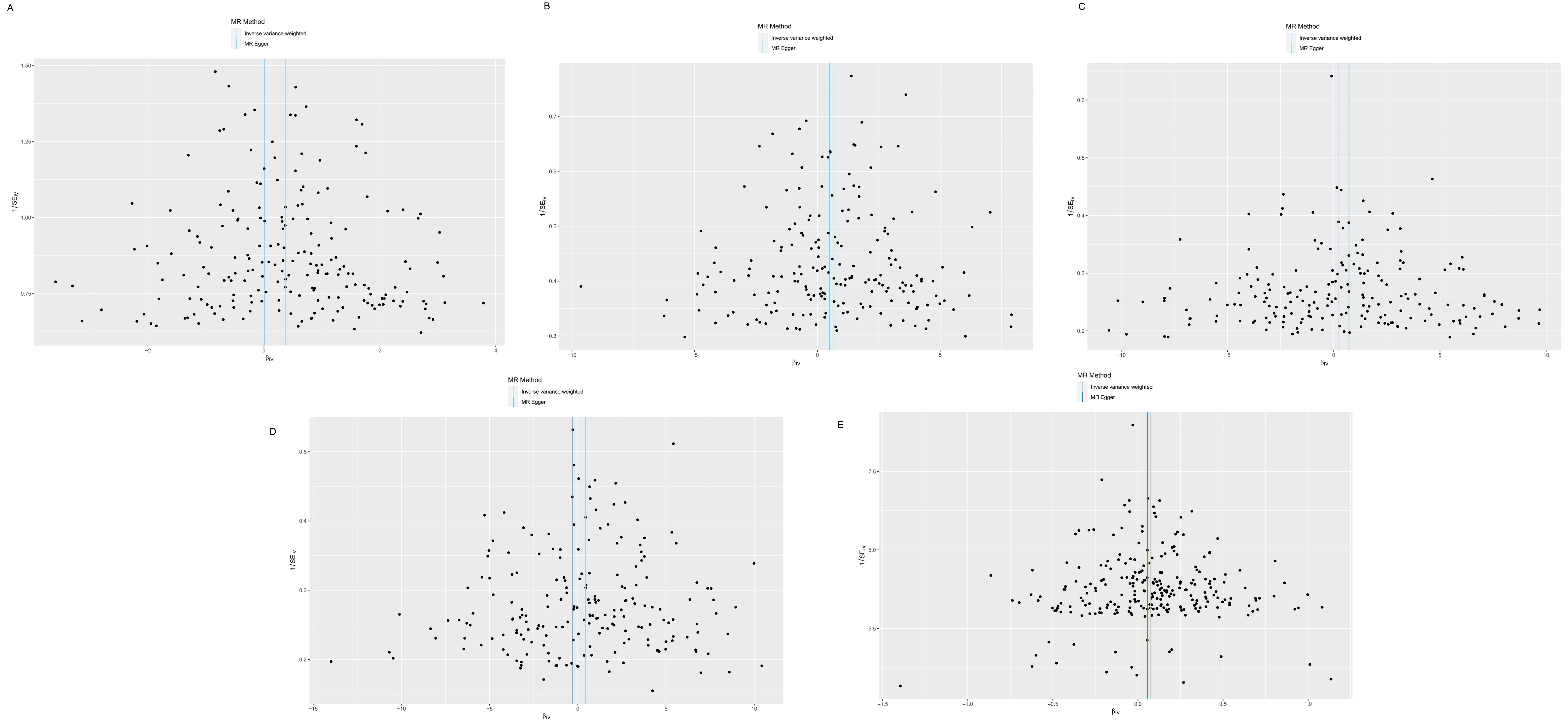
A
MR Method
Inverse variance weighted
MR Egger
1/SE_IV
β_IV
B
MR Method
Inverse variance weighted
MR Egger
1/SE_IV
β_IV
C
MR Method
Inverse variance weighted
MR Egger
1/SE_IV
β_IV
D
MR Method
Inverse variance weighted
MR Egger
1/SE_IV
β_IV
E
MR Method
Inverse variance weighted
MR Egger
1/SE_IV
β_IV
