# Supplementary material for: Externalizing traits: Shared causalities for COVID-19 and Alzheimer's dementia using Mendelian randomization analysis
Source: PNAS Nexus. 2023 Jun 15;2(6):pgad198. doi: 10.1093/pnasnexus/pgad198 (PMC10287533; doi:10.1093/pnasnexus/pgad198)
Supplement: pgad198_Supplementary_Data [file pgad198_supplementary_data.zip › PNASNEXUS-PNASNEXUS-2022-00978RR-s02.pdf]

Figure S2. MR leave-one-out sensitivity analysis for the effect of externalizing traits on COVID-19.

Leave-one-out analysis: each row represents an MR analysis of externalizing traits on COVID-19 using all instruments except for the SNP associated with externalizing traits listed on the y-axis. The point represents the beta with that SNP removed, and the line represents the 95% confidence interval.

COVID-19: Coronavirus disease 2019; MR: Mendelian randomization.

Figure S3. MR leave-one-out sensitivity analysis for the effect of externalizing traits on Hospitalized COVID-19.

Leave-one-out analysis: each row represents an MR analysis of externalizing traits on hospitalized COVID-19 using all instruments except for the SNP associated with externalizing traits listed on the y-axis. The point represents the beta with that SNP removed, and the line represents the 95% confidence interval.

COVID-19: Coronavirus disease 2019; MR: Mendelian randomization.

Figure S4. MR leave-one-out sensitivity analysis for the effect of externalizing traits on COVID-19 without hospitalization.

Leave-one-out analysis: each row represents an MR analysis of externalizing traits on COVID-19 without hospitalization using all instruments except for the SNP associated with externalizing traits listed on the y-axis. The point represents the beta with that SNP removed, and the line represents the 95% confidence interval.

COVID-19: Coronavirus disease 2019; MR: Mendelian randomization.

Figure S5. MR leave-one-out sensitivity analysis for the effect of externalizing traits on Severe COVID-19.

Leave-one-out analysis: each row represents an MR analysis of externalizing traits on severe COVID-19 using all instruments except for the SNP associated with externalizing traits listed on the y-axis. The point represents the beta with that SNP removed, and the line represents the 95% confidence interval.

COVID-19: Coronavirus disease 2019; MR: Mendelian randomization.

Figure S6. MR leave-one-out sensitivity analysis for the effect of externalizing traits on AD.

Leave-one-out analysis: each row represents an MR analysis of externalizing traits on Alzheimer's dementia using all instruments except for the SNP associated with externalizing traits listed on the y-axis. The point represents the beta with that SNP removed, and the line represents the 95% confidence interval.

AD: Alzheimer's dementia; MR: Mendelian randomization.

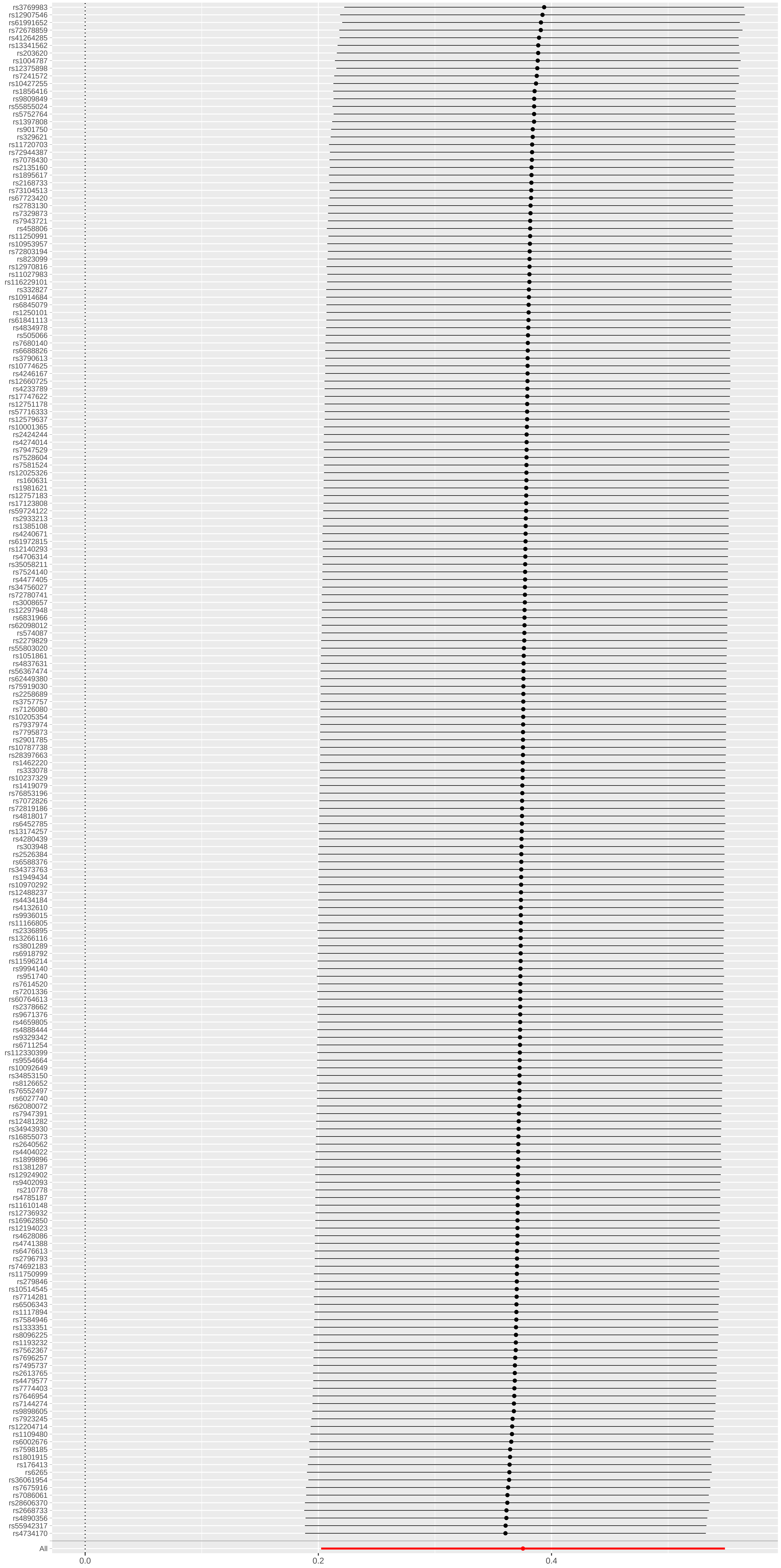

Figure S2. Leave−one−out sensitivity analysis for externalizing traits on COVID-19

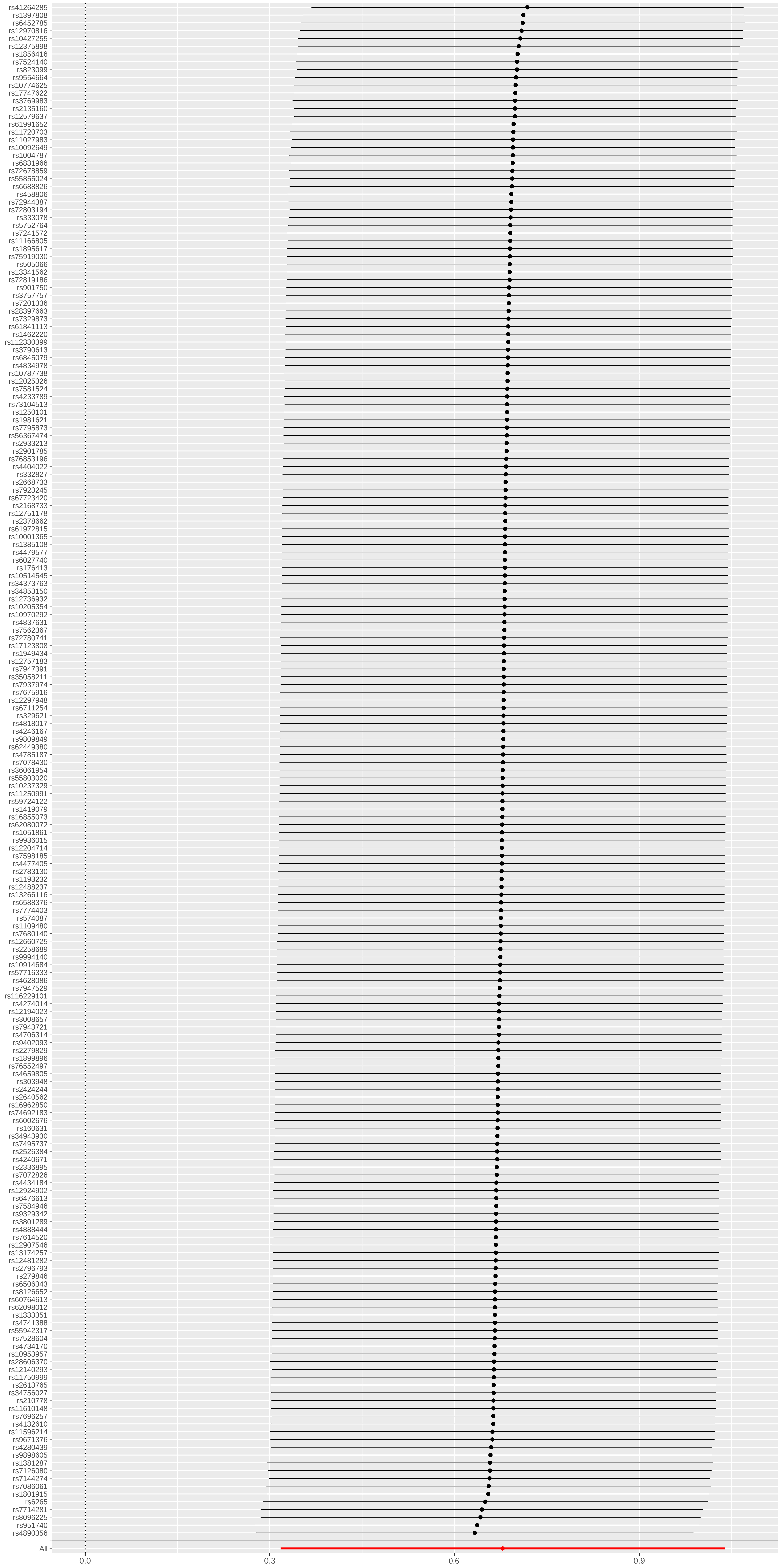

Figure S3. MR leave−one−out sensitivity analysis for externalizing traits on Hospitalized COVID-19

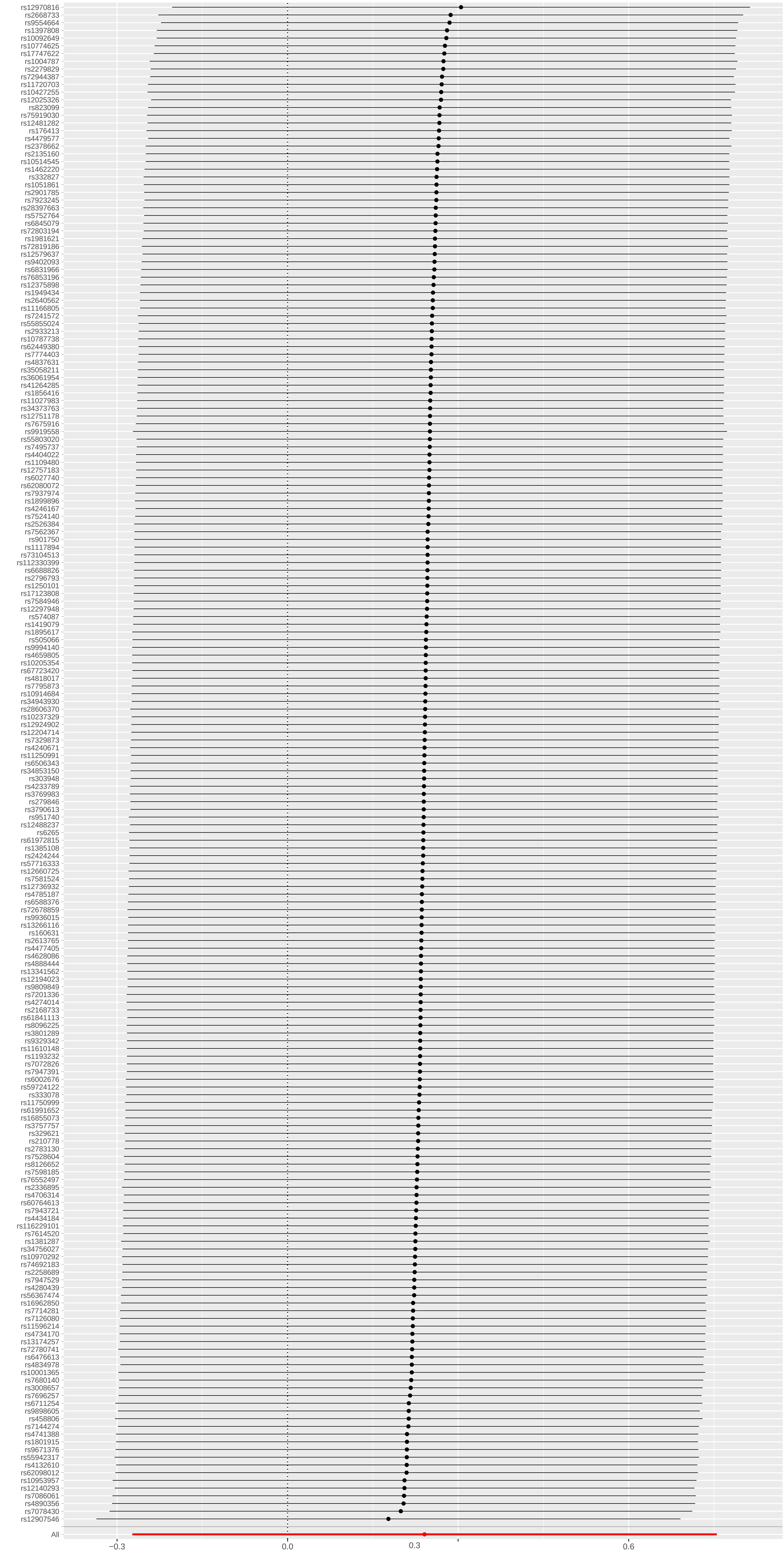

Figure S4. MR leave−one−out sensitivity analysis for externalizing traits on COVID-19 without hospitalization

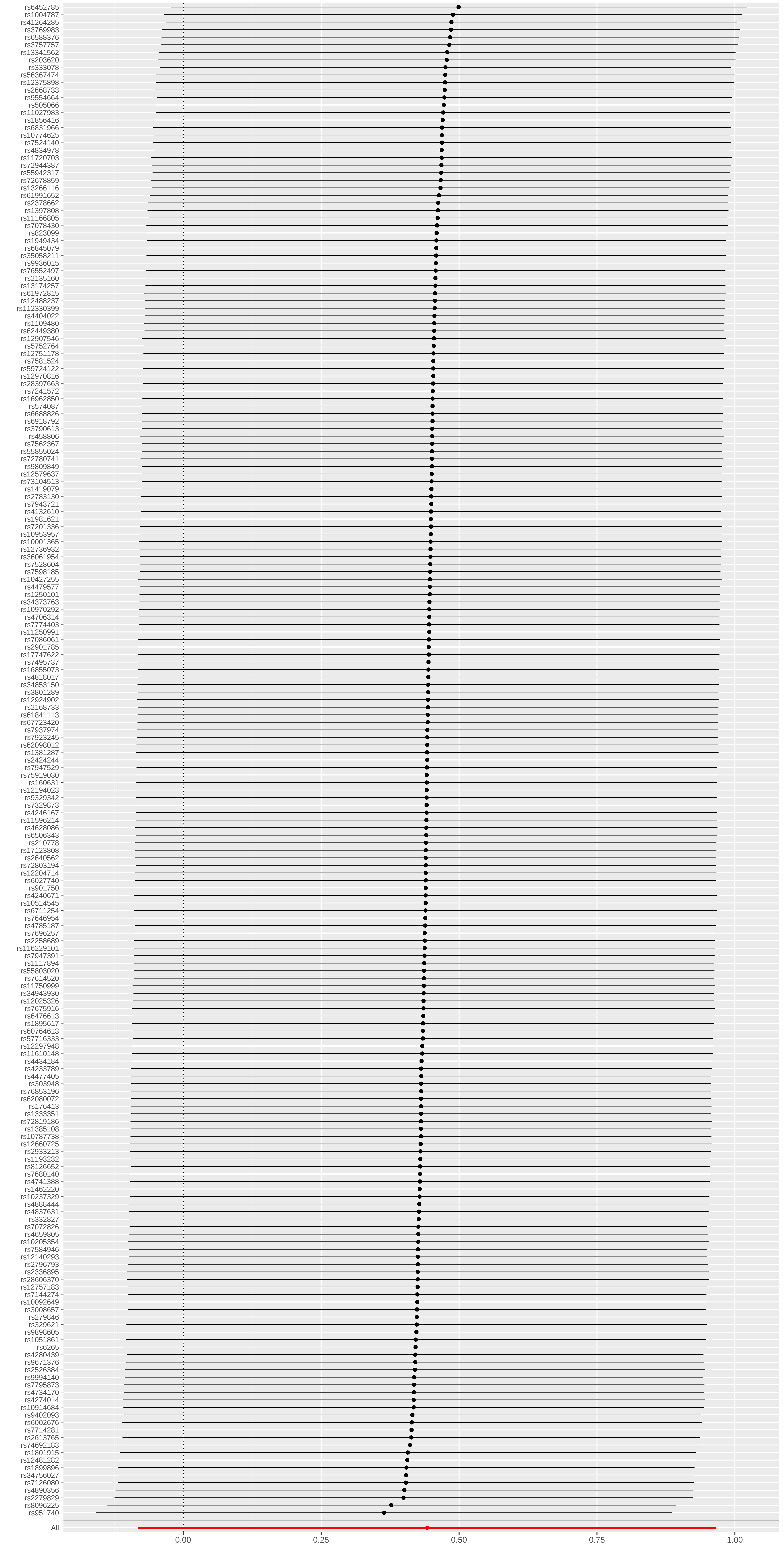

Figure S5. Leave−one−out sensitivity analysis for externalizing traits on Severe COVID-19

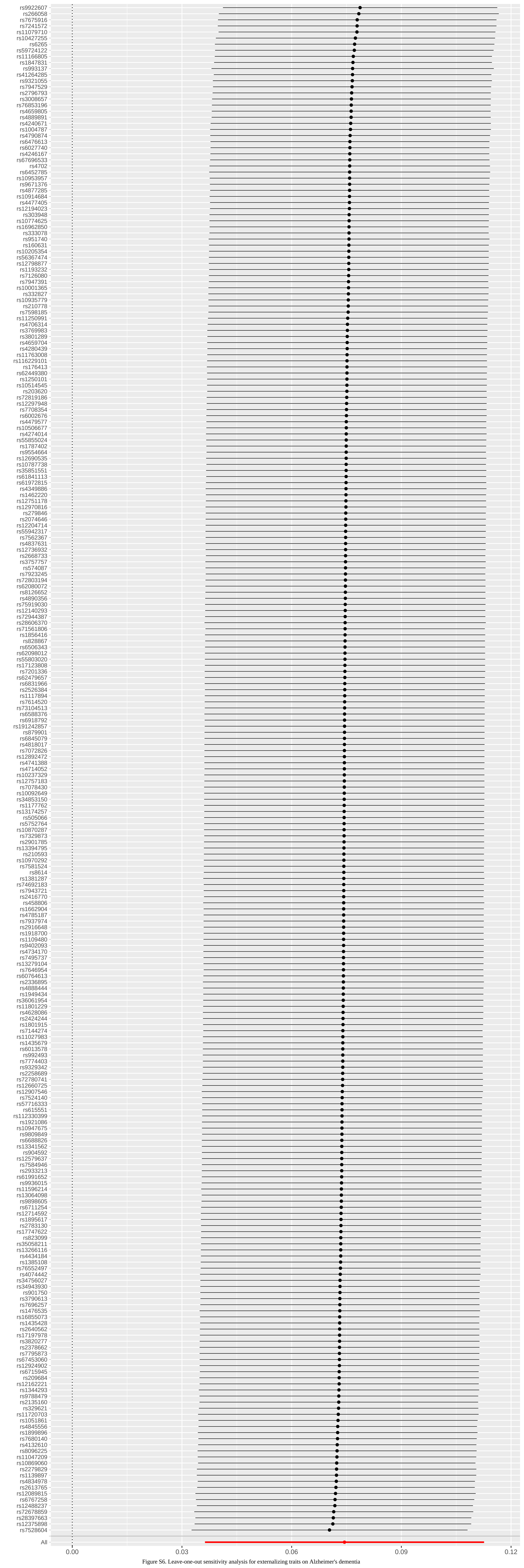

Figure S6. Leave-one-out sensitivity analysis for externalizing traits on Alzheimer's dementia
